# Integration of the Opportunity‐Ability‐Motivation behavior change framework into a coaching‐based WHO Safe Childbirth Checklist program in India

**DOI:** 10.1002/ijgo.12542

**Published:** 2018-06-20

**Authors:** Lisa R. Hirschhorn, Margaret Krasne, Jenny Maisonneuve, Nabihah Kara, Tapan Kalita, Natalie Henrich, Darpan Rana, Pinki Maji, Megan M. Delaney, Rebecca Firestone, Narender Sharma, Vishwajeet Kumar, Atul A. Gawande, Katherine E.A. Semrau

**Affiliations:** ^1^ Northwestern Feinberg School of Medicine Chicago IL USA; ^2^ Harvard Medical School Boston MA USA; ^3^ Ariadne Labs Brigham and Women's Hospital Harvard T.H. Chan School of Public Health Boston MA USA; ^4^ Population Services International Lucknow India; ^5^ Populations Services International Washington DC USA; ^6^ Community Empowerment Lab Lucknow India; ^7^ Department of Surgery Brigham and Women's Hospital Boston MA USA; ^8^ Department of Health Policy and Management Harvard T.H. Chan School of Public Health Boston MA USA; ^9^ Division of Global Health Equity Brigham and Women's Hospital Boston MA USA; ^10^ Department of Medicine Harvard Medical School Boston MA USA

**Keywords:** Behavior change, Childbirth, Coaching, Facility‐based delivery, India, Opportunity‐Ability‐Motivation framework, Quality of care, WHO Safe Childbirth Checklist

## Abstract

**Objective:**

To evaluate whether integration of the Opportunity‐Ability‐Motivation plus Supplies (OAMS) framework into coaching improved the delivery of essential birth practices in a low‐resource setting.

**Methods:**

This prospective mixed‐methods study used routine coaching visit data obtained from the first eight intervention facilities of the BetterBirth trial in Uttar Pradesh, India, between December 19, 2014, and October 21, 2015. The 8‐month intervention was peer coaching that integrated the OAMS framework to support uptake of the WHO Safe Childbirth Checklist. Descriptive statistics were used to measure nonadherence to essential birth practices. The frequency and accuracy of coaches’ coding of barriers and the appropriateness of chosen resolution strategies to measure feasibility, acceptability, and fidelity of using OAMS, were assessed.

**Results:**

Coaches observed 666 deliveries, including 12 602 practices. Overall, essential practice nonadherence decreased from 15.6% (262/1675 practices observed) to 4.5% (4/88 practices) (*P*<0.001). Of the 1048 barriers identified, opportunity (556 [53.1%]) and motivation (287 [27.4%]) were the most frequently reported categories; the frequency of both decreased over time (*P*=0.003 and *P*<0.001, respectively). The coaches appropriately categorized 930 (99.8%) of 932 barriers and provided an appropriate strategy for 800 (85.8%). The commonest reason for unaddressed barriers was lack of coaching opportunities.

**Conclusion:**

Successful integration of OAMS framework into delivery attendant coaching enabled coaches to rapidly diagnose barriers to practice adherence and develop responsive strategies.

**ClinicalTrials.gov:**

NCT2148952 (WHO Universal Trial Number: U11111‐1315‐647).

## Abstract 2

Using a modified behavior change framework, peer‐coaches were able to identify barriers to the provision of essential delivery practices and design problem‐solving interventions.

## 1 INTRODUCTION

Childbirth‐related mortality remains a major cause of suffering, globally, with 350 000 maternal and 3.1 million neonatal deaths annually.^1^, ^2^ Essential birth practices (EBPs) reduce maternal and neonatal morbidity and mortality; however, care providers do not employ these practices widely and consistently.^3^ Although policy efforts have improved women's access to facility‐based delivery, poor quality of care remains problematic in many resource‐constrained settings.^4^, ^5^

To address the quality gap in maternal and neonatal care during facility‐based delivery, WHO and other stakeholders created the WHO Safe Childbirth Checklist (SCC), a 28‐item tool consisting of EBPs associated with improved maternal, fetal, and neonatal outcomes.^6^, ^7^, ^8^ The SCC is organized to drive change at four critical moments (or pause points): on admission, before delivery, within 1 hour after delivery, and before discharge. Initial studies have demonstrated an association between SCC use and improved adherence to EBPs.^9^, ^10^, ^11^

Evidence from quality‐improvement initiatives has shown the importance of integrated interventions to change both provider behavior and the healthcare system. When done well, supportive supervision, clinical mentorship, and coaching can be effective in changing provider behavior in a variety of settings, increasing the rate of skill transfer or adoption and generating more sustained improvement in performance than training alone.^12^, ^13^, ^14^

To maximize the impact of the SCC, a coaching‐based implementation program (the BetterBirth program^15^) was designed, and—based on behavior change literature from multiple fields—the Opportunity‐Ability‐Motivation (OAM) framework was integrated into this coaching strategy.^16^ The OAM framework, initially developed for understanding individual consumer behavior,^17^ postulates that barriers to and facilitators of behavior change operate within three domains: opportunity, ability, and motivation. Researchers in a number of fields including public health have adopted the OAM framework.^17^, ^18^ Given the prevalence of challenges associated with supplies and equipment in many resource‐constrained settings, in the present study the OAM framework was adapted by dividing opportunity into supply‐related and other opportunity‐related barriers (Opportunity‐Ability‐Motivation‐Supplies [OAMS]).

During the BetterBirth trial, routine coach‐reported data were collected to study whether coaches correctly and effectively applied the OAMS framework in diagnosing and addressing barriers to EBP performance among delivery attendants. The present study used data obtained from the first eight intervention facilities to evaluate whether integration of the OAMS framework into the BetterBirth coaching approach was feasible and acceptable; this was measured by the uptake and correct application by the coaches to rapidly diagnose barriers to practice adherence and develop responsive strategies.

## 2 MATERIALS AND METHODS

The present study was a prospective mixed‐methods study leveraging data collected by coaches as part of their work in the BetterBirth trial—a cluster‐randomized controlled trial that was designed to test the effectiveness of a coaching‐based implementation of the WHO SCC in Uttar Pradesh, India's most populous state. Uttar Pradesh has persistently high maternal and neonatal mortality rates.^19^ The present study included all data collected by coaches between December 19, 2014, and October 21, 2015, in the first eight intervention sites. The facilities included in the BetterBirth trial provided labor and delivery services 24 hours a day on 7 days each week, had a minimum of 1000 deliveries per year, and employed at least three delivery attendants.^15^ The study protocol was approved by the ethics review committees of the following institutions: Community Empowerment Lab, Lucknow, India; Jawaharlal Nehru Medical College, Belgaum, India; Harvard T.H. Chan School of Public Health, Boston, MA, USA; Population Services International; and WHO. The study was also approved by the Indian Council of Medical Research.

Trained coaches (nurses) and coach team leaders (physicians or public health professionals) engaged in three main tasks at the individual and facility levels: (1) encouraging behavior change; (2) observing, documenting, and feeding back information about EBP performance and SCC use; and (3) joint problem‐solving to resolve barriers to behavior change.^20^ The coaching model was multilevel, collaborative, and person‐centered. The coaches visited each intervention facility during an 8‐month period with decreasing frequency, from twice weekly to monthly. The program did not provide supplies (except paper copies of the SCC), equipment, or monetary incentives; the coaches did not provide direct clinical skills building.

At each observation of a delivery attendant providing care, the coach completed an Observation Tool to Inform Support (OTIS) by recording the performance (or nonperformance) of each EBP. If, after prompting, a delivery attendant did not perform an EBP, the coach documented at least one barrier obstructing the delivery attendant's performance of the EBP and categorized that barrier according to the OAMS framework as follows.^7^, ^20^ Opportunity‐related barriers were defined as environmental or contextual factors beyond an individual's control (excluding supplies or equipment); examples include inadequate time because multiple women were in labor or women were already delivering when coming into the hospital. Ability‐related barriers were defined as gaps in an individual's skills or knowledge; examples include not knowing when to measure blood pressure or how to prepare the delivery tray. Motivation‐related barriers were defined as a lack of interest or belief in the value of a given practice; examples include an unwillingness to take the body temperature or the belief that the SCC is not important. Supplies‐related barriers were defined as the absence of physical supplies or equipment required for the performance of a given EBP; examples include lack of oxytocin or a blood pressure device and no water for hand washing.

In late February 2015, 2 months after initiation of the BetterBirth trial at the first study site, the coaches began using a coach support tool to record a brief narrative description of new barriers prioritized during a visit and any unresolved barriers that had been prioritized in earlier visits. The coaches recorded an OAMS category for each barrier and at least one specific coaching strategy they applied to resolve the barrier. These strategies could include a direct intervention, escalating to management to address system barriers such as facility stock‐outs, deferring to the next visit if the delivery attendant was too busy or no patient was available for observing any behavior change, continuing interventions into the next coaching visit if no change was seen, or abandoning if the delivery attendant would no longer be working in labor and delivery. The coach support tool could have multiple entries if the coach prioritized more than one EBP challenge to address at a given visit or a challenge persisted over time. The quotes presented in this paper are written verbatim from the coaches’ notes with abbreviations explained where needed.

In a quantitative analysis, eligible OTIS data were used to calculate the rates of nonadherence to EBPs, the application of the framework (acceptability), and the frequency of coach‐reported barriers in each OAMS category overall and for individual EBPs at five time points; admission, pre‐delivery, the post‐delivery pause point divided into two coach observation periods to reflect the different practices required immediately after delivery and within 1 hour, and at discharge. Changes in EBP nonadherence over time were assessed by comparing the nonadherence frequencies in different 2‐week coaching periods using Poisson log‐linear regression. The analyses were completed using SPSS version 23 (IBM, Armonk, NY, USA), Excel version 15.15 (Microsoft, Redmond, WA, USA), and Stata version 14.2 (StataCorp, College Station, TX, USA).

In addition, a qualitative analysis was conducted to understand the feasibility and fidelity of using the coaching approach and learn about barrier classification and strategies to address identified barriers. For this purpose, a subset of the coach support tool entries completed between February 19 and June 30, 2015, were translated from Hindi. Free‐text descriptions of the barriers and coaching strategies were extracted. One author (MK) coded these descriptions for barrier category and for fidelity and feasibility of the applied coaching strategy (assessing whether the OAMS category of the strategy matched the OAMS category of the barrier); a second author (LRH) reviewed the coding. The team resolved any differences with discussion.

Owing to considerable overlap between strategies designed to address individual‐level barriers (ability and motivation) and system‐related barriers (opportunity and supplies), the barriers and strategies were grouped into these two broader categories for the qualitative analysis. Entries with multiple barrier categories were coded as appropriately addressed if at least one of the coaching strategies matched with at least one of the barrier categories. Commonly used strategies employed by BetterBirth coaches were summarized.

Each facility's leadership provided facility‐level consent for participation in the BetterBirth trial and for introduction of the BetterBirth program, which included the feedback of coaching and programmatic data. Verbal informed consent and contact information was obtained from each woman (or her surrogate) enrolled for follow‐up in the BetterBirth trial. At the beginning of the coaching intervention, each facility and each delivery attendant formally agreed to participate in the BetterBirth program as a quality improvement initiative. The coaches accompanied the delivery attendants during their work shifts, and the documentation of practices during patient care activities at the facilities was part of the programmatic monitoring and coaching activities. The coaches collected no patient identifiers and all delivery attendant identifiers were removed before analysis for the present paper. The patients enrolled in the trial for follow‐up were not the same patients who were observed during coaching.

## 3 RESULTS

Across the eight intervention facilities, 46 delivery attendants received coaching from a median of 2.5 coaches per site (10 individual coaches). During the 8 months, the coaches observed 666 deliveries at one or more pause points, documenting 12 602 EBPs across 1352 SCC pause points (see Table S1 for more details). Overall, the nonadherence rate for the EBPs documented in OTIS was 7.9% (997/12 602), with variation in nonadherence by pause point: at facility admission, 10.1% (268/2664) of EBPs were missed; 10.8% (414/3848) were missed just prior to delivery; 3.4% (43/1270) were missed at the time of delivery, 6.4% (196/3040) were missed within the first hour postpartum, and 7.1% (127/1780) were missed at discharge (Fig. S1). The rates of nonadherence to specific EBPs ranged from 0.0% (evaluation of the neonate's breathing) to higher rates such as 39.5% (taking the mother's temperature before delivery) (data not shown).

For the 997 EBPs that were not completed, the coaches showed high acceptability of the framework and recorded 1048 barriers. Individual‐level barriers (motivation and ability) and system‐level barriers (supplies and opportunity) accounted for 32.7% (n=343) and 67.3% (n=705) of all barriers reported, respectively. Opportunity was the most frequently reported barrier category (556 [53.1%]), followed by motivation (287 [27.4%]), supplies (149 [14.2%]), and ability (56 [5.3%]). The relative distribution of the barrier categories varied between the pause points. For example, motivation was a more common barrier to EBP performance during admission (95/268 [35.4%]) and 1 hour after delivery (87/196 [44.4%]) than just before delivery (67/414 [16.2%]) and during delivery (2/43 [4.7%]).

**Figure 1:**
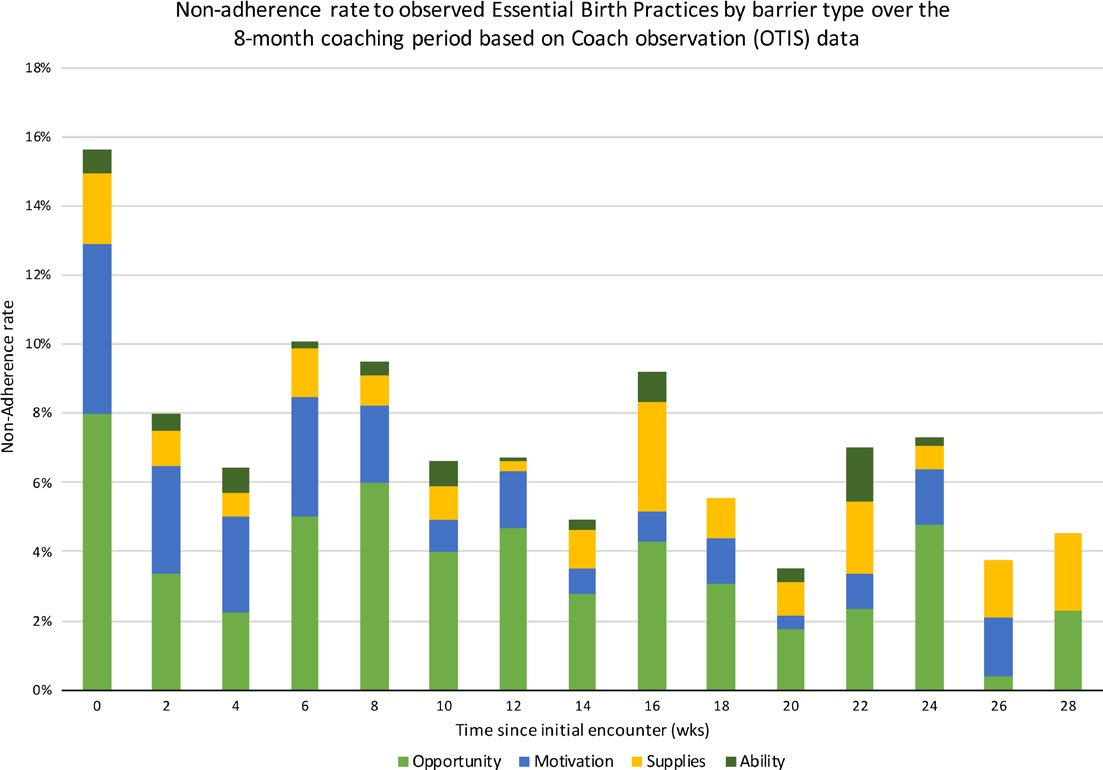
Rate of nonadherence to essential birth practices stratified by barrier type during an 8‐month coaching period (based on coach observation data collected with the Observation Tool to Inform Support).

According to OTIS data, the nonadherence rate decreased from 15.6% (262/1675) in the beginning of coaching to 4.5% (4/88) in the final 2 weeks of coaching (*P*<0.001) (Fig. 1). The rate of EBP nonadherence attributable to opportunity barriers decreased by an average of 3.3% relative to each previous 2‐week period (*P*=0.003), and the rate of EBP nonadherence attributable to motivation barriers decreased by an average of 9.1% (*P*<0.001). The rates of EBP nonadherence attributable to ability and supplies barriers did not change significantly over time.

The coach support tool captured qualitative coaching data on 29 delivery attendants across the eight sites. From a total of 955 reports of EBP nonadherence, four were excluded because the barrier was resolved prior to the next visit, 14 because no mother was present at the next visit (and therefore a previously identified barrier could not be evaluated), and five because written descriptions of the barrier were missing. For the remaining 932 EBPs not performed, 130 (13.9%) had an opportunity barrier identified, 578 (62.0%) an ability barrier, 308 (33.0%) a motivation barrier, and 97 (10.4%) a supplies barrier (Table 1). Nearly all (930 [99.8%]) the barriers not performed had been categorized appropriately by the coaches, reflecting high feasibility and fidelity. For 800 (85.8%) of the 932 EBPs not performed, the coaches implemented strategies that corresponded to at least one of the barrier categories recorded. Thirty cases involved strategies that addressed two barrier categories. If the barrier was not addressed, the coaches most commonly cited a lack of coaching opportunity as the reason (Table 2).

**Table 1:** Classification of, and response to, barriers among 932 non‐completed essential birth practices documented in the coach support tool

Coach‐coded barrier	Frequency^a^	Example
Opportunity	130 (13.9)	“Because there is a lot of work, BA says is not able to take BP at PP4.”
Ability	578 (62.0)	“BA does not know why it is necessary to check for bleeding.”
Motivation	308 (33.0)	“BA does not want to use the SCC.”
Supplies	97 (10.4)	“Baby linen has run out in the supply.”
System level (opportunity and/or supply)	223 (23.9)	“No water supply in labor room. BA said when water supply will be fixed she will be able to wash hand.”
Individual level (ability and/or motivation)	851 (91.3)	“Does not know importance of taking BP, BA does not want to take BP either.”
Combination of system and individual level	146 (15.7)	“Thermometer was not available in the labor room. Does not know importance of taking temperature.”
		“Oxytocin is not in supply. BA does not understand the importance of keeping oxytocin prepared.”

Abbreviations: BA, birth attendant; BP, blood pressure; PP4, pause point 4 (before discharge); SCC, WHO Safe Childbirth Checklist.

a Values are given as number (percentage).

**Table 2:** Response to barriers among 932 non‐completed essential birth practices documented in the coach support tool

Response	No. (%)
Strategy implemented	
Strategy was responsive to described barrier	800 (85.8)
Strategy did not match any of the described barriers	21 (2.3)
No strategy implemented	
No patient available, delivery attendant too busy, or delivery attendant no longer continuing in maternity service	92 (9.8)
Follow‐up behavior was only observed	19 (2.0)

The coaches recorded many different strategies to help delivery attendants resolve barriers to performing EBPs (Table 3). These strategies ranged from telling a story to motivate an individual delivery attendant to involving a facility administrator to address a supply stock‐out (Box 1). By way of example, in one busy facility, delivery attendants explained that they lacked sufficient time to prepare a delivery tray for each mother. The coaches suggested assigning a worker who was not a delivery attendant to prepare the trays. Implementation of this strategy ensured the completion of EBPs related to delivery supplies and gave delivery attendants the opportunity to focus on other EBPs. Other examples included the coach using the SCC and other motivation techniques such as storytelling to encourage delivery attendants to integrate the EBPs to meet national standards and save lives. When delivery attendants successfully overcame a barrier, the coaches also celebrated the behavior change to encourage sustaining the improvement while moving on to other challenges.

**Table 3:** Illustrative examples of BA's barriers to adhering to essential birth practices, coaching strategies delivered, and identified next steps in the BetterBirth trial

Barrier type	Essential birth practice	Barrier	Coaching strategy	Identified next steps
Individual level (ability or motivation)	Danger signs explained to mother/companion	“BA does not properly know. She is not properly paying attention while explaining danger signs.”	“After demonstrating to BA by role‐playing, she understood. Just after, another delivery arrived so she explained danger signs to the woman's relatives.”	“I will observe BA again if she is explaining danger signs to each patient.”
	Taking maternal BP	“BA does not know importance of taking maternal BP.”	“With help of [SCC]…motivated to take mother BP. BA took BP.”	“Continue coaching. Next visit I will observe BA.”
	Oxytocin administered right after delivery	“Lack of proper knowledge.”	“I have prepared to role‐play according to [SCC].”	“Continue coaching.”
	Oxytocin administered right after delivery	“Stock‐out.”	“Through [coach team leader], talked to MOIC [head of the facility] about oxytocin and when it will come into supply. … said that until oxytocin comes into supply, BA will order oxytocin from outside.”	“Continue coaching. Today there was no case so could not observe. Next visit I will observe.”
System level (opportunity or supply)	Handwashing	“No water in labor room.”	“When asked, BA said we should talk with [head of facility] … [who] suggested holding a meeting to understand what needs to be done. BA was still using water from bucket and water got finished before delivery and she could not wash hands.”	“[Coach supervisor] should request a meeting to resolve the water supply issue.”
	Taking the maternal temperature	“Thermometer is not in the labor room”	“Spoke to pharmacist to make thermometer available.”	“Thermometer has arrived in the [labor room]. Next visit I will observe BA to see if she is taking every patient's temperature or not.”
Both individual and system level	Taking the maternal BP	“Mercury BP machine did not have battery inside.”	“After discussing with BA, found out that BP machine's battery had died. Motivated BA to order new battery and told BA to take BP using the [SCC] as well.”	“It is not in the BA's habits to take BP yet so coaching is still necessary.”
		“BA also does not know the importance of BP.”	“BA took BP.”	—

Abbreviations: BA, birth attendant; BP, blood pressure; MOIC: Medical Officer in Charge (Physician in charge of the facility); SCC, WHO Safe Childbirth Checklist**.**

BOX 1 **Summary of frequently used coach‐delivered strategies by barrier type**

1

**Table:** 

Opportunity Suggest task‐sharing for supply preparation to free up time to deliver other EBPs Suggest delivery attendants use hand sanitizer if there is no running water or no time to wash hands before deliveries Have water buckets brought in for delivery attendants to wash hands when water is otherwise unavailable Prepare oxytocin before each delivery so it is immediately available after delivery Supplies Advocate with administrators or pharmacists about obtaining missing supply Motivation Motivate delivery attendant to use the SCC by referring to the SCC and national guidelines Motivate delivery attendant by using stories, motivational videos, and SBA guidelines Show data from OTIS heatmap to encourage change from red or yellow to green Acknowledge and “appreciate” delivery attendant when she performs EBP well Ability Explain importance of specific EBP Ask senior clinical staff to help educate and provide clinical coaching Use old completed SCC to explain proper practice Have delivery attendant perform SCC demonstration

Abbreviations: EBP, essential birth practice; OTIS, Observation Tool to Inform Support; SBA, skilled birth attendant; SCC, WHO Safe Childbirth Checklist.

## 4 DISCUSSION

The OAMS framework was a feasible and acceptable structure for the coaching‐based implementation of the WHO SCC. The coaches were able to categorize barriers to EBP adherence using the framework with high fidelity and develop coaching strategies that appropriately reflected and addressed these underlying barriers. This coaching approach, incorporating the OAMS framework, was associated with an increase in adherence to the observed EBPs over 8 months, although there was no change in morbidity and mortality.^21^, ^22^

Using the OAMS framework also enabled tracking change in the types of barriers coaches faced over time. The percent of nonadherence attributable to motivation barriers decreased during the 8 months of the study—a change consistent with the focus of coaching on individual behavior change through observation, motivation, and feedback rather than on clinical skills mentoring.^20^, ^21^ Similarly, the percent of EBP nonadherence attributable to opportunity barriers decreased over time, whereas the percent attributable to supplies did not. This pattern is consistent with the coaching strategies used to address supplies barriers, which involved problem‐solving to shift tasks appropriately among staff or to reorganize available supplies as opposed to providing new supplies, an intervention not included in the BetterBirth Program. Additionally, in many instances the coaches had to address overlap within and between individual‐level barriers (motivation and ability) and system‐level barriers (supplies and opportunity). In these situations, the coaches often prioritized one barrier to avoid overwhelming the delivery attendants with too many proposed changes simultaneously.

The OAMS framework offered a concrete way of teaching coaches and coach team leaders how to recognize, develop, and share quality improvement strategies in different facility contexts.^20^ Initial and later refresher coaching trainings were practical, incorporating role‐playing and active learning to ensure coaches’ understanding of the categories. The coach team leaders also used OAMS to direct their supportive supervision of coaches and to offer feedback on the quality of the coaches’ documentation. Moreover, the coaches used the OAMS framework to collaborate to improve coaching across the program by sharing strategies, brainstorming new strategies, and mentoring new coaches on necessary skills associated with identifying and addressing barriers.

The present study contains a number of limitations. The analyses included program data routinely captured through a quantitative tool (OTIS) and convenience‐sample data captured through a qualitative tool (the coach support tool) rather than data collected by independent observers. Moreover, it was not possible to confirm that the barriers described by the coaches accurately represented the reality of care provision. Social or professional pressures may have led some coaches to report changes in EBP adherence they felt program leadership expected. To improve the validity of reporting, the coach team leaders provided ongoing supportive supervision, including on‐site coaching of coaches, double observation, and double coding of observations, to improve accuracy. A Hawthorne effect could also account for some of the changes in EBP adherence over time, particularly as the coaching relationships developed.

The time periods covered by OTIS and the coach support tool differ, resulting in differences in the relative proportion of data from OTIS forms and coach support tools between sites. Therefore, data from these two sources were not directly compared. It was also not possible to follow specific delivery attendants over time to directly measure the effectiveness of the implemented strategies. Finally, because the study lacked a control group, the observed changes in EBP adherence cannot be conclusively attributed to the coaching‐based implementation of the SCC. However, no other maternal and newborn health quality improvement interventions took place in these eight facilities during the study period. Future work should include the evaluation of different strategies implemented by coaches for specific barriers to identify which approaches are most effective in changing and sustaining behavior.

In conclusion, integration of the OAMS behavior change framework into the coaching‐based implementation of the WHO SCC was acceptable, feasible and facilitated coaches’ correct categorization of barriers and their development of appropriately responsive strategies to address these barriers. The use of OAMS‐informed coaching was associated with an increase in adherence to EBPs.^21^ By contrast, supervision—as currently delivered in some settings—is not always associated with higher quality of care.^23^ The present findings support the potential for coaching informed by the OAMS framework in conjunction with the WHO SCC to inspire behavior change in front‐line providers and encourage them to use the skills they have gained through pre‐ and in‐service training. These findings make this framework‐based coaching an important tool to consider for programs that aim to strengthen the quality of care through the performance of evidence‐based practices.

## AUTHOR CONTRIBUTIONS

LRH, MK, and KEAS contributed to the design and planning of the study, data analysis, and writing and revising the manuscript. JM contributed to conducting the study and data collection, data analysis, and writing and revising the manuscript. NK contributed to the design and planning of the study, conducting the study and data collection, data analysis, and revising the manuscript. TK contributed to conducting the study and data collection, and revising the manuscript. NH contributed to data analysis and revising the manuscript. DR, PM, MMD, NS, and VK contributed to conducting the study, data collection, and revising the manuscript. RF contributed to the design and planning of the study, conducting the study and data collection, and revising the manuscript. AAG contributed to the design and planning of the study, and revising the manuscript.

## CONFLICTS OF INTEREST

AAG has received royalties for books and essays, including publications on improving the quality and delivery of health care using checklists. The authors have no other conflicts of interest.

## Supporting information


**Figure S1.** Rate of nonadherence to essential birth practices by observation point in the first eight facilities participating in the BetterBirth trial (based on coach observation data collected with the Observation Tool to Inform Support).

**Table S1.** Distribution of observations made by coaches and data collection tools used in the first eight facilities participating in the BetterBirth trial.
