# Supplementary figures and images for: Integration of the Opportunity‐Ability‐Motivation behavior change framework into a coaching‐based WHO Safe Childbirth Checklist program in India
Source: Int J Gynaecol Obstet. 2018 Jun 20;142(3):321–8. doi: 10.1002/ijgo.12542 (PMC6099329; doi:10.1002/ijgo.12542)

## Slide 1
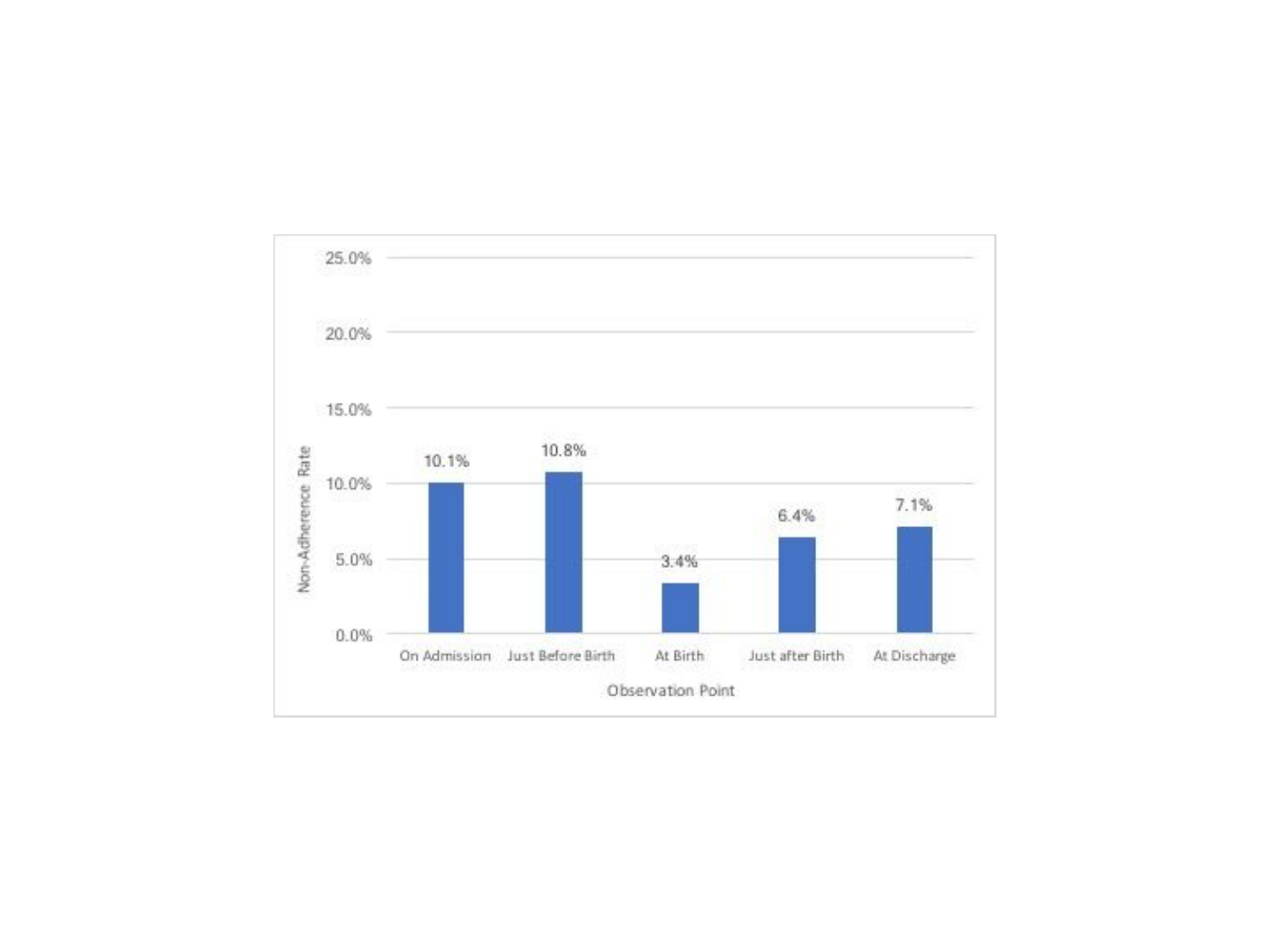

Supplement: Supplementary file 1 — Figure S1. Rate of nonadherence to essential birth practices by observation point in the first eight facilities participating in the BetterBirth trial (based on coach observation data collected with the Observation Tool to Inform Support). [file IJGO-142-321-s001.pptx]
