# Supplementary material for: Integration of the Opportunity‐Ability‐Motivation behavior change framework into a coaching‐based WHO Safe Childbirth Checklist program in India
Source: Int J Gynaecol Obstet. 2018 Jun 20;142(3):321–8. doi: 10.1002/ijgo.12542 (PMC6099329; doi:10.1002/ijgo.12542)
Supplement: Supplementary file 2 — Table S1. Distribution of observations made by coaches and data collection tools used in the first eight facilities participating in the BetterBirth trial. [file IJGO-142-321-s002.docx]

**Table S1 Distribution of observations made by Coaches and data collection tools used in the first 8 facilities of the BetterBirth trial.EBP: Essential Birth Practices.**

|  |  |  | **Observation Tool to Inform Support (OTIS)** | | | | **Coach Support Tool (CST)** | |
| --- | --- | --- | --- | --- | --- | --- | --- | --- |
| **Site** | **Coaches with ≥1 visit**  **(n)** | **Birth Attendants**  **(n)** | **Births Observed**  **(n)** | **% total** | **Observation points completed**  **(n)** | **% total** | **Reported EBP challenges (n)** | **% total** |
| 1 | 2 | 8 | 33 | 5.0% | 64 | 4.7% | 164 | 17.2% |
| 2 | 2 | 6 | 99 | 14.9% | 174 | 12.9% | 47 | 4.9% |
| 3 | 2 | 8 | 118 | 17.7% | 227 | 16.8% | 95 | 9.9% |
| 4 | 2 | 6 | 86 | 12.9% | 196 | 14.5% | 264 | 27.6% |
| 5 | 3 | 4 | 63 | 9.5% | 149 | 11.0% | 184 | 19.3% |
| 6 | 3 | 4 | 59 | 8.9% | 123 | 9.1% | 63 | 6.6% |
| 7 | 4 | 4 | 100 | 15.0% | 198 | 14.6% | 79 | 8.3% |
| 8 | 5 | 6 | 108 | 16.2% | 221 | 16.3% | 59 | 6.2% |
| **Total** | 10 individual Coaches | 46  individual birth attendants | 666 | 100% | 1352 | 100% | 955 | 100% |
